# Determination of Flavonoids Compounds of Three Species and Different Harvesting Periods in *Crataegi folium* Based on LC-MS/MS

**DOI:** 10.3390/molecules26061602

**Published:** 2021-03-13

**Authors:** Ya-Ping Guo, Hong Yang, Ya-Li Wang, Xiao-Xiang Chen, Ke Zhang, Yan-Li Wang, Yi-Fan Sun, Jian Huang, Lu Yang, Jin-Hui Wang

**Affiliations:** 1 School of Pharmacy, Harbin Medical University, Harbin 150000, China; guoyaping_0816@163.com (Y.-P.G.); yanghonghyd2016@163.com (H.Y.); wangyanli@hrbmu.edu.cn (Y.-L.W.); 17644049614@163.com (Y.-F.S.); wangjinhui@hrbmu.edu.cn (J.-H.W.); 2 Key Laboratory of Xinjiang Phytomedicine Resource and Utilization, Ministry of Education, College of Pharmacy, Shihezi University, Shihezi 832002, China; wyl182em@163.com (Y.-L.W.); chen852110@163.com (X.-X.C.); tcm_zk@163.com (K.Z.); 3 Shenzhen Honghui BioPharmaceutical Co. Ltd., Shenzhen 518118, China; 4 Key Laboratory of Forest Resources and Utilization in Xinjiang of National Forestry and Grassland Administration, Xinjiang Academy of Forestry, Key Laboratory of Fruit Tree Species Breeding and Cultivation in Xinjiang, Urumqi 830052, China

**Keywords:** *Crataegus songorica*, *Crataegus altaica*, *Crataegus kansuensis*, LC-MS/MS, flavonoids

## Abstract

*Crataegi folium* have been used as medicinal and food materials worldwide due to its pharmacological activities. Although the leaves of *Crataegus songorica* (CS), *Crataegus altaica* (CA) and *Crataegus kansuensis* (CK) have rich resources in Xinjiang, China, they can not provide insights into edible and medicinal aspects. Few reports are available on the qualitative and quantitative analysis of flavonoids compounds of their leaves. Therefore, it is necessary to develop efficient methods to determine qualitative and quantitative flavonoids compounds in leaves of CS, CA and CK. In the study, 28 unique compounds were identified in CS versus CK by qualitative analysis. The validated quantitative method was employed to determine the content of eight flavonoids of the leaves of CS, CA and CK within 6 min. The total content of eight flavonoids was 7.8–15.1 mg/g, 0.1–9.1 mg/g and 4.8–10.7 mg/g in the leaves of CS, CA and CK respectively. Besides, the best harvesting periods of the three species were from 17th to 26th September for CS, from 30th September to 15th October for CA and CK. The validated and time-saving method was successfully implemented for the analysis of the content of eight flavonoids compounds in CS, CA and CK for the first time.

## 1. Introduction

Hawthorn is a deciduous tree of Crataegus in family Rosaceae. Seventeen species of Crataegus are recorded in Flora Reipublicae Popularis Sinicae (FRPS). A great deal of attention has recently been paid to *Crataegus pinnatifida* Bge and varieties of Shanlihong (*Crataegus pinnatifida* Bge. var. major N.E.Br.), *Crataegus brettschneideri* (Fu hawthorn) and *Crataegus scabrifolia* (Yun nan hawthorn) in China [1]. However, *Crataegus songorica* (CS), *Crataegus altaica* (CA) and *Crataegus kansuensis* (CK) have hardly any reports. *Crataegi folium* have been widely applied for medicinal and food materials in China and European countries [2]. Their leaves play an important role in improving digestion [3], promoting blood circulation [4,5], immune functions [6] and sugar and lipid metabolism [7] in China for a long period. Some flavonoids compounds such as vitexin-2”-*O*-glucoside, vitexin-2”-*O*-rhamnoside, vitexin, rutin, hyperoside, isoquercitrin, quercetin and epicatechinin have been isolated from the leaves of *Crataegus pinnatifida* [8,9,10] as the main active compounds, which exhibited a wide range of pharmacological activities including cardiovascular diseases [11,12,13], antihypertensive [14] and anti-inflammatory [15], free radical scavenging [16], exercise tolerance and improved the symptoms of fatigue and breathing shortness [5,17]. In the recent study, these flavonoids compounds are analyzed by a high-performance liquid chromatographic (HPLC) [9,18,19], capillary zone electrophoresis (CZE) method [20], high-speed counter-current chromatography (HSCCC) [21] and ultra-performance liquid chromatography electrospray ionization tandem mass spectrometric (UPLC–ESI–MS) methods [22], which have been carried out the pharmacokinetics of vitexin-2”-*O*-rhamnoside and vitexin-4”-*O*-glucoside that expressed they were effective compounds.

CS, CA and CK are primarily three hawthorn species in Xinjiang (China) [23], previous literature mostly focused on ornamental tree species and rootstocks and breeding materials for cultivated hawthorn. However, there are no reports of quantitative and qualitative analysis of flavonoids compounds in their leaves. Therefore, the study aimed to develop a quantitative and qualitative method based on LC-MS/MS for simultaneous determination of 8 flavonoids compounds (rutin, quercetin, hyperoside, isoquercetin, vitexin-2”-*O*-glucoside, vitexin-2”-*O*-rhamnoside, vitexin and epicatechin) from different harvesting periods of the leaves of CS, CA and CK. Furthermore, similarities and differences of the 8 flavonoids compounds between different species and harvesting periods were clarified according to the principal component analysis (PCA) and orthogonal projections to latent structures discriminant analysis (OPLS−DA). Collectively, our study laid the foundation for the comprehensive utilization and subsequent research of the leaves of CS, CA and CK.

## 2. Results and Discussion

### 2.1. The Results of Qualitative Analysis

#### 2.1.1. Identification of Unique Compounds in Three Species of *Crataegi folium*

Chemspider and Progenesis Metascope (SDF) databases were used to identify unknown compounds. Additionally, rutin, quercetin, hyperoside, isoquercetin, vitexin-2”-*O*-glucoside, vitexin-2”-*O*-rhamnoside, vitexin and epicatechin as a reference were determined. According to the high score, high fragmentation score and mass error ≤5 ppm, just a few compounds had a mass error of 5–10 ppm and adducts ([M-H]^−^, [M-H_2_O-H]^−^ and [M+FA-H]^−^) were screened, and 216 compounds in total were identified in the leaves of CS, CA and CK in Table S1, and among them, there were 97 flavonoids, 13 triterpenoids, 31 phenylpropanoids and so on. Based on the Progenesis QI statistical analysis had a fold range of ≥2 and analysis of variance (ANOVA) *p*-value of ≤0.05 and VIP in S-plot of ≥1. A maximum false discovery rate (FDR)-adjusted *q* value threshold of 0.01 was used for unique compounds.

**Figure 1:**
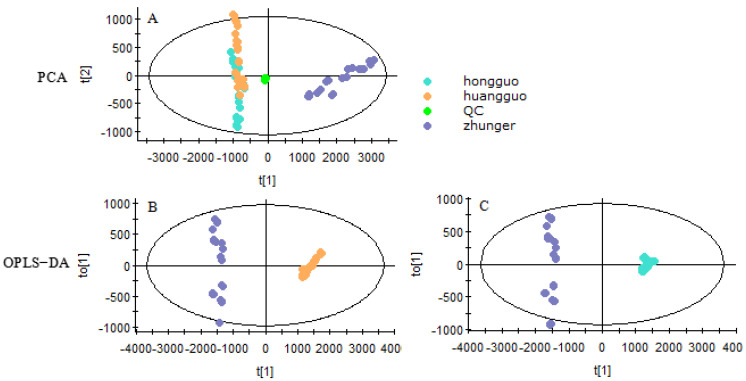
The statistical analysis of identified compounds of the score plot from principal component analysis (PCA) (**A**) and orthogonal projections to latent structures discriminant analysis (OPLS−DA) model with Hotelling’s 95% confidence ellipse from *Crataegi folium* of *Crataegus songorica* (CS) (zhunger) vs. *Crataegus altaica* (CA) (huanggguo) (**B**) and CS vs. *Crataegus kansuensis* (CK) (hongguo) (**C**).

The PCA and OPLS−DA plot of 216 identified compounds in the leaves of CS, CA and CK were fitted by EZInfo 3.0. Besides, R^2^X and Q^2^ (cum) were usually used to evaluate the quality of the PCA model, both R^2^X and Q^2^ (cum) were higher, the quality of the PCA model was better. The R^2^X and Q^2^ of the PCA model of CS, CK and CA were 0.990 and 0.956 respectively, which showed the PCA model fit well. As shown in Figure 1A, the QC sample formed a tight cluster, proving the reliability of the acquired data, CA and CK were separated from CS. To look for unique compounds, OPLS−DA was generated, and R^2^X, R^2^Y, and Q^2^ (cum) were mostly used to estimate the quality of the OPLS−DA model. Both R^2^X, R^2^Y and Q^2^ (cum) were higher, the OPLS−DA model was better. Moreover, as for the OPLS−DA model of CS versus CA, R^2^X, R^2^Y and Q^2^ were 0.911, 0.989 and 0.988, and CS versus CK were 0.907, 0.995 and 0.994 respectively that showed these models with fit good. As shown in Figure 1B,C, either CS versus CA or CS versus CK was apart well, which expressed they had a remarkable difference.

**Figure 2:**
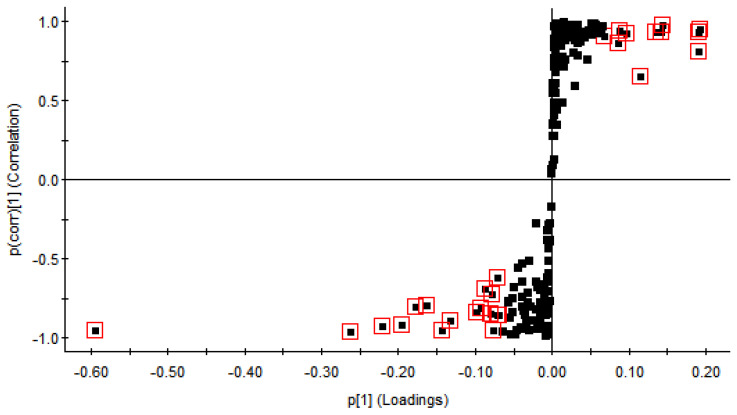
The S-plot of identified compounds from OPLS−DA of CS vs. CK, labeled with a variable importance plot (VIP) >1.

To relate the compound and different species the scatter plot (S-plot) and variable importance plot (VIP) were used to confidence intervals from the OPLS−DA model. The S-plot shows a graphical interpretation of the covariance and the correlation between the loading variables and the predictive score *t* [1,24]. The *p* [1] axis defines the magnitude of each variable in X, while the *p* (corr) [1] axis represents the reliability of each variable in X, thus, the X-variables farthest from the origin combine high influence with high reliability in the model [25] and the further a marker is from the origin, the greater it is their contribution to the variance between these groups [26]. Taking CS versus CK as an example, an S-Plot of OPLS−DA was fitted, and the compounds of VIP ≥ 1 were picked, which was revealed in the red box in Figure 2, 28 unique compounds were screened in Table 1.

Normalized abundances were used to calculate relative content of 28 unique compounds in Figure S2. Among them, rutin, 6-*C*-glucoside-8-*C*-xylsoyl apigenin, vitexin-6”-*O*-acetyl, vitexin, vitexin-2”-*O*-glucoside, vitexin-3”-*O*-acetyl, 7-*O*-rhamnogalactoside quercetin vitexin-2”-*O*-rhamnoside and so on were reported in *Crataegus pinnatifida* Bge, besides, rutin, vitexin, vitexin-2”-*O*-glucoside and vitexin-2”-*O*-rhamnoside were compared with the reference. As shown in Figure S2, compared with total content of 28 unique compounds, we found the leaves of CS were always higher than CK in different harvesting periods, and the content of vitexin-2”-*O*-rhamnoside was highest in the leaves of CS. Moreover, we discovered the constitution of compounds were notable difference in CS and CK, which could be used to distinguish different species of *Crataegi folium.* In addition, the variation trend of 28 unique compounds in different harvesting periods can be a reference to optimize harvesting periods.

#### 2.1.2. The Main Fragmentation Patterns of Representative Flavonoids Compounds

There were various skeleton types of flavonoids in *Crataegi folium*, in this study the main types obtained were vitexin-type, quercetin-type and epicatechin-type. A strong excimer ion peak of [M-H]^−^ was acquired.

**Figure 3:**
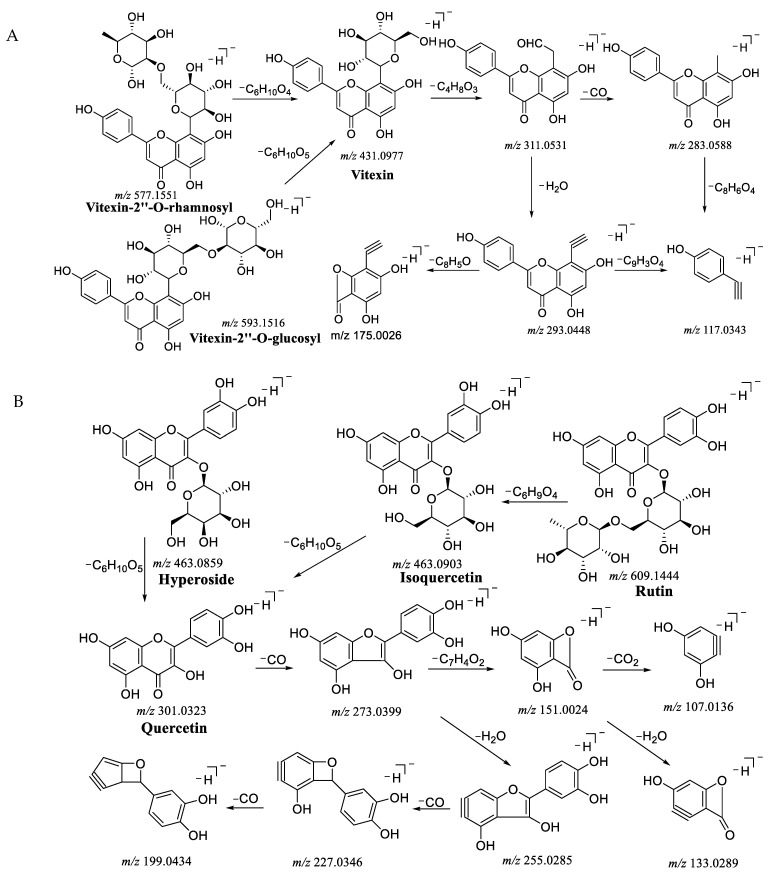

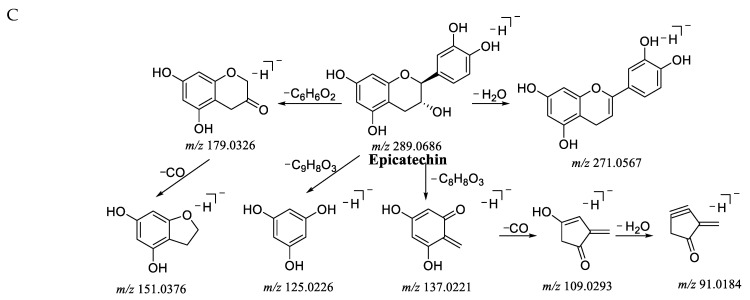
Mass spectrograms of representative flavonoids of different types. (**A**) Vitexin-type flavonoids, (**B**) Quercetin-type flavonoids and (**C**) Epicatechin-type flavonoids.

The vitexin-type were mostly from a C_4_H_8_O_3_ loss and generated [M-C_4_H_8_O_3_-H]^−^ (*m/z* 311.0531) in Figure 3A, and then one H_2_O loss and obtained [M-C_4_H_8_O_3_-H_2_O-H]^−^ (*m/z* 293.0448), another CO loss and acquired [M-C_4_H_8_O_3_-CO-H]^−^ (*m/z* 283.0588), after that, a common ion *m/z* 177.0343 was observed. Finally, a C_8_H_5_O loss from *m/z* 293.0448 and generated the ion *m/z* 175.0026. Furthermore, vitexin-2”-*O*-rhamnoside ([M-H]^−^, *m/z* 577.1551) lost one rhamnose (C_6_H_10_O_4_) and obtained vitexin, in the same way, vitexin-2”-*O*-glucoside ([M-H]^−^, *m/z* 593.1516) lost one glucose (C_6_H_10_O_5_) and generated vitexin.

The quercetin-type were primarily from a CO loss and developed [M-CO-H]^−^ (*m/z* 273.0799) shown in Figure 3B, and then a H_2_O loss and obtained [M-CO-H_2_O-H]^−^ (*m/z* 255.0285), due to the loss of CO, respectively, the base peak ions were [M-CO-CO-H_2_O-H]^−^ (*m/z* 227.0346) and [M-CO-CO-CO-H_2_O-H]^−^ (*m/z* 199.0434). On the other way, a C_7_H_4_O_2_ lost and formed [M-CO-C_7_H_4_O_2_-H]^−^ (*m/z* 151.0024), because of the loss of one H_2_O or CO_2_, the peak ions were [M-CO-C_7_H_4_O_2_-H_2_O-H]^−^ (*m/z* 133.0289) or [M-CO-C_7_H_4_O_2_-CO_2_-H]^−^ (*m/z* 107.0136). In addition, one rhamnose (C_6_H_9_O_4_) lost from rutin ([M-H]^−^, *m/z* 609.1444) and performed isoquercetin ([M-H]^−^, *m/z* 463.0903), and then one glucose (C_6_H_10_O_5_) lost from isoquercetin and hyperoside and conducted quercetin ([M-H]^−^, *m/z* 301.0323).

As shown in Figure 3C, epicatechin-type were preeminently from a H_2_O loss and carried out fragment peak [M-H_2_O-H]^−^ (*m/z* 271.0567), resulted from the loss of C_6_H_6_O_2_, C_9_H_8_O_3_, and C_8_H_8_O_3_, the fragment ions were [M-C_6_H_6_O_2_-H]^−^ (*m/z* 179.0326), [M-C_9_H_8_O_3_-H]^−^ (*m/z* 125.0226) and [M-C_8_H_8_O_3_-H]^−^ (*m/z* 137.0221), independently. Subsequently, with one CO loss, [M-C_6_H_6_O_2_-CO-H]^−^ (*m/z* 151.0376) was generated. According to the loss of CO and H_2_O step by step, a series of fragment peaks [M-C_8_H_8_O_3_-CO-H]^−^ (*m/z* 109.0293) and [M-C_8_H_8_O_3_-CO-H_2_O-H]^−^ (*m/z* 91.0184) originated.

### 2.2. The Results of the Quantitative Analysis

#### 2.2.1. Validated Method Data

**Figure 4:**
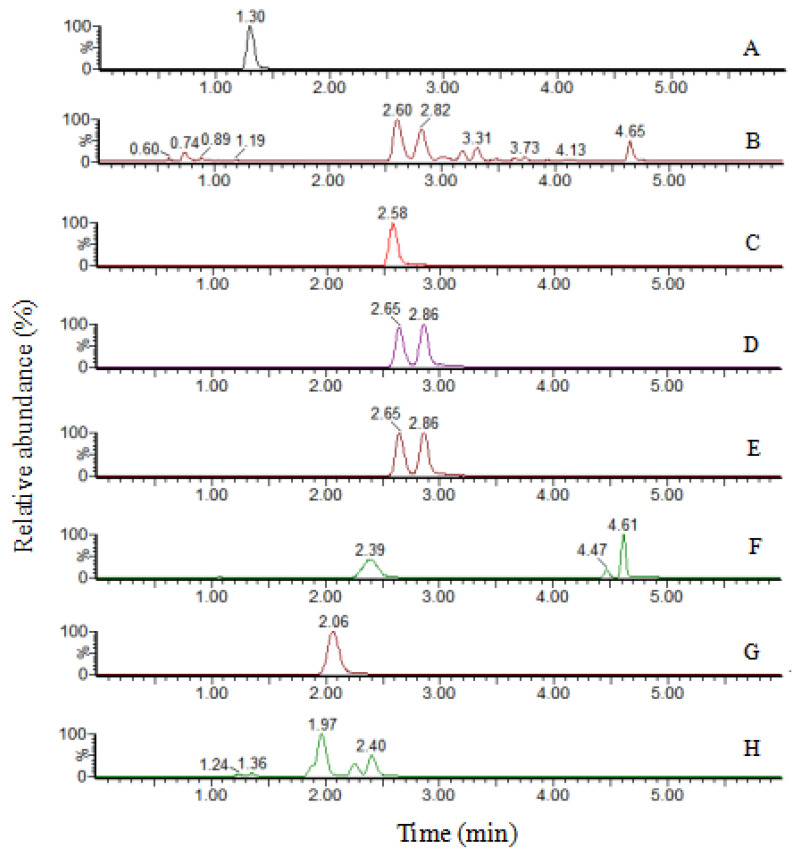
UPLC-MS/MS chromatograms of eight flavonoids of the leaves in CS. (**A**) epicatechin (1.30 min); (**B**) quercetin (4.65 min); (**C**) vitexin (2.58 min); (**D**) isoquercetin (2.86 min); (**E**) hyperoside (2.65 min); (**F**) vitexin-2”-*O*-rhamnoside (2.39 min); (**G**) vitexin-2”-*O*-glucoside (2.06 min) and (**H**) rutin (2.40 min).

UPLC-MS/MS chromatograms of eight flavonoids of the leaves in CS were shown in Figure 4. Mixed standards were prepared in concentrations ranging from 0.001 to 5 μg/mL. Standard curves were carried out with coefficients of determination (*r*) higher than 0.999. The accuracy, repeatability, and recovery were calculated from the mean and relative standard deviation (RSD) of six replicates, the RSD of accuracy was at the range from 1.26 to 1.99%, and the repeatability was 1.41–3.78%. The recoveries were at the range of 95–105%, the mean recoveries at 98.1–100.9%. The detailed results were shown in Table 2. The validated quantitative method was supposed that is appropriate for the determination of the eight flavonoids standards of the leaves of different harvesting period of CS, CA and CK.

#### 2.2.2. The Content of Eight Flavonoids Compounds in Different Species of *Crataegi folium*

**Figure 5:**
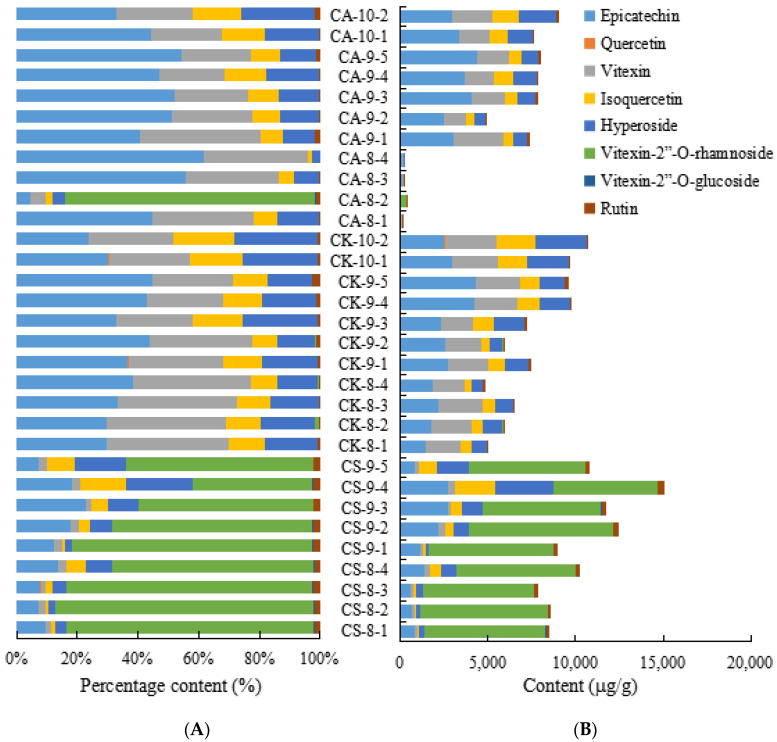
The content of eight flavonoids compounds of leaves of CS, CA and CK. (**A**) Percentage stacked column of eight flavonoids compounds. (**B**) Stacked column of eight flavonoids compounds.

The detailed content of eight flavonoids compounds of the leaves of CS, CA and CK were shown in Table S2, to elucidate the variation tendency of eight flavonoids compounds in different species, their stack columns were generated in Figure 5. We found all eight flavonoids compounds could be detected in the leaves of CS, CA and CK. Furthermore, vitexin-2”-*O*-rhamnoside, hyperoside, isoquercetin and epicatechin in the leaves of CS were the main contribution compounds, and most of them, the content of vitexin-2”-*O*-rhamnoside was the highest (6.0–8.2 mg/g) (Table S2), which was a main active compound in *Crataegus pinnatifida* Bge. In addition, it is worth noting that vitexin-2”-*O*-rhamnoside had a higher content in CS than CA and CK in Figure 5B.

Additionally, the content of hyperoside, isoquercetin, vitexin and epicatechin was higher than that of the other flavonoids in the leaves of CK, but vitexin-2”-*O*-glucoside was not detected at all. Interestingly, the varied trend of hyperoside, isoquercetin, vitexin and epicatechin presented an analogical trend in the leaves of CA as shown in Figure 5A. These results expressed that there is a similarity between CA and CK to a certain degree.

**Figure 6:**
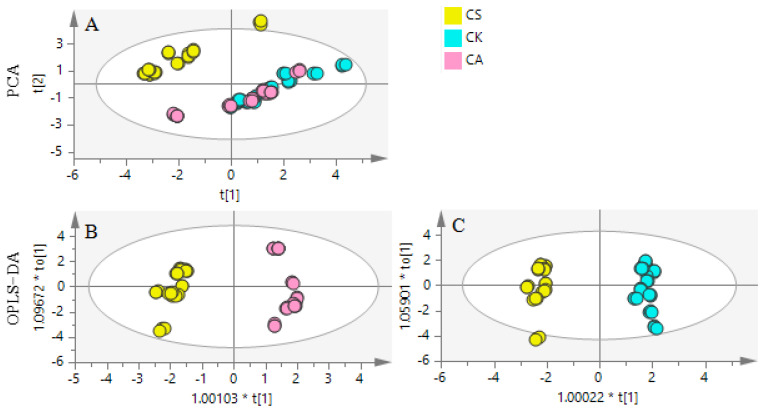
The statistical analysis of the content of eight flavonoids compounds score plot from PCA and OPLS−DA model with Hotelling’s 95% confidence ellipse of *Crataegi folium*. (**A**) PCA of CS, CA and CK; (**B**) OPLS−DA of CS vs. CA and (**C**) OPLS−DA of CS vs. CK.

To elucidate the distribution characteristics in different species and further look for more available resources, the PCA was applied to illustrate the correlations between the contents of flavonoids compounds of different species of hawthorn leaves. The R^2^X and Q^2^ of the PCA model of CS, CK and CA were 0.971 and 0.840 separately, which performed the PCA model fit good. The closer the two species lie on the plot, the more similar they are in the content of flavonoids compounds. On the contrary, a species that is distant from the others can have significantly different content of compounds. The triplicate LC-MS/MS analyses of each sample are overlaid on the plot, indicating good repeatability of the chemical analysis. As shown in Figure 6A, according to the PCA plot, the samples could be divided into two groups, CK and CA were grouped in the same cluster, and CS were separated from CK and CA that indicated the leaves of CS had a remarkable difference with CK and CA.

**Figure 7:**
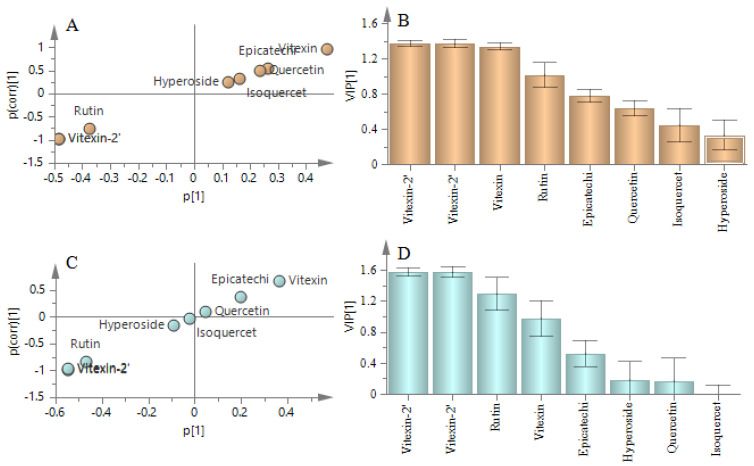
The statistical analysis of the content of eight flavonoids compounds S-plot from OPLS−DA of CS vs. CK (**A**), CS vs. CA (**C**) and variable importance plot (VIP) with 95% jack-knifed confidence intervals of CS vs. CK (**B**) and CS vs. CA (**D**).

To explore the difference between the leaves of CS and CK/CA, the score plot from the OPLS−DA model was conducted. In terms of the model of CS and CA, R^2^X, R^2^Y and Q^2^ were 0.857, 0.973 and 0.972 and for the model of CS and CK they were 0.882, 0.989 and 0.988 respectively, which showed these models with a goodness of fit. The OPLS−DA scores plot (Figure 6B,C) showed a good separation between CS and CA, CS and CK, which expressed the difference of the content of flavonoids compounds in different species. Combined S-plot and VIP, by filtering the compounds of VIP >1, we discovered rutin, vitexin-2”-*O*-glucoside, vitexin-2”-*O*-rhamnoside and vitexin were the grater contribution to the variance between CS vs. CK (Figure 7A,B). Vitexin-2”-*O*-rhamnoside, vitexin-2”-*O*-glucoside and rutin were the main contributions between CS vs. CA (Figure 7C,D). As a result, vitexin-2”-*O*-rhamnoside, vitexin-2”-*O*-glucoside and rutin were the major markers as crucial active compounds in the leaves of CS, CA and CK compared with other species [27]. What is more, CS was suitable for further exploration.

#### 2.2.3. Comparative Analysis of Different Harvesting Periods of *Crataegi folium*

**Figure 8:**
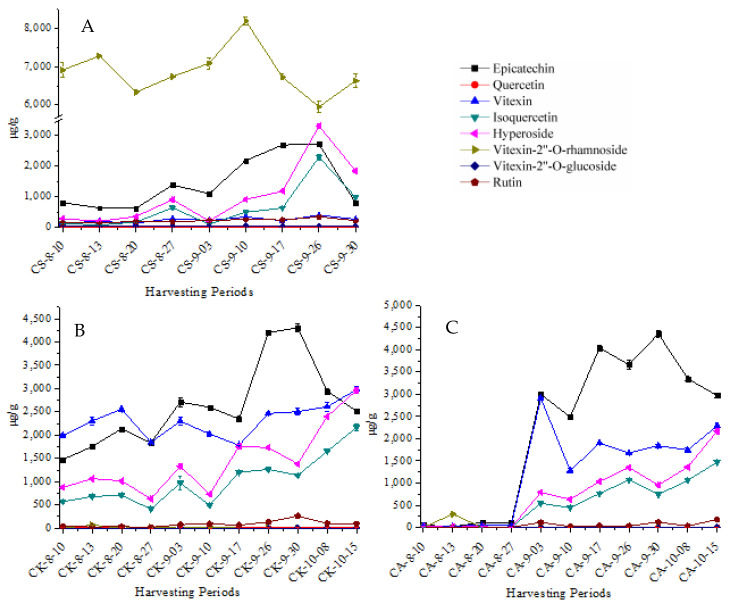
Changes in the content of eight flavonoids in leaves of CS (**A**), CK (**B**) and CA (**C**) during different harvesting. periods (*n* = 3).

Figure 8A showed the various levels of eight flavonoids in the leaves of CS. The contents of epicatechin, hyperoside and isoquercetin remained the same trend in the whole harvesting period and increased progressively in September until reaching the highest levels on 26th September, and decreased after that, however, the content of vitexin-2”-*O*-rhamnoside reached the highest level (8.2 mg/g) in 10th September and reduced to the lowest level (6.0 mg/g) in 26th September and rose slowly by the end of the harvesting period. Quercetin and vitexin-2”-*O*-glucoside always stayed stable in the whole harvesting period, which may be a result from the low content. Moreover, vitexin and rutin achieved the highest levels on 26th September and decreased after that which were consistent with epicatechin, hyperoside and isoquercetin. For most of the content of all compounds, the vitexin-2”-*O*-rhamnoside was the highest one. In a word, the best harvesting period should be from 17th to 26th September of the leaves of CS.

Figure 8B presented the changing patterns in the content of eight flavonoids in the leaves of CK, hyperoside and isoquercetin remained the same trend in whole harvesting period, before 17th September, epicatechin and vitexin kept accordance in CK, but epicatechin gained the highest levels on 30th September, hyperoside, isoquercetin and vitexin increased the highest in 15th October because of correlation between contents of quercetin derivatives. The accumulation of flavonoids in leaves was commonly suggested to be sensitive to the environment. A negative correlation has been found between the contents of quercetin derivatives in tomato leaves and harvesting temperature [28]. The other compounds remained constant in the whole harvesting period. Consequently, the best harvesting period should be in from 30th September to 15th October of the leaves of CK.

Figure 8C expressed the varied trend the content of eight flavonoids in the leaves of CA, 27th August was a key date for CA, after that, the content of epicatechin, vitexin, hyperoside and isoquercetin of CA increasing sharply in early September, and then vitexin attained the highest content in 3rd September, and decreased quickly in 10th September, after that increasing slowly, until the end of harvesting period reattained the second-highest level. After 27th August, epicatechin continued to grow, until it increased up to the highest level on 30th September and then reduced slowly. The trends of hyperoside and isoquercetin were in accordance with CK. In general, the change of the content of eight flavonoids in CK and CA were similar. Therefore, the best harvesting period should be from 30th September to 15th October of the leaves of CK, and CK was an alternative species to study the content of eight flavonoids in different parts in the next research.

**Figure 9:**
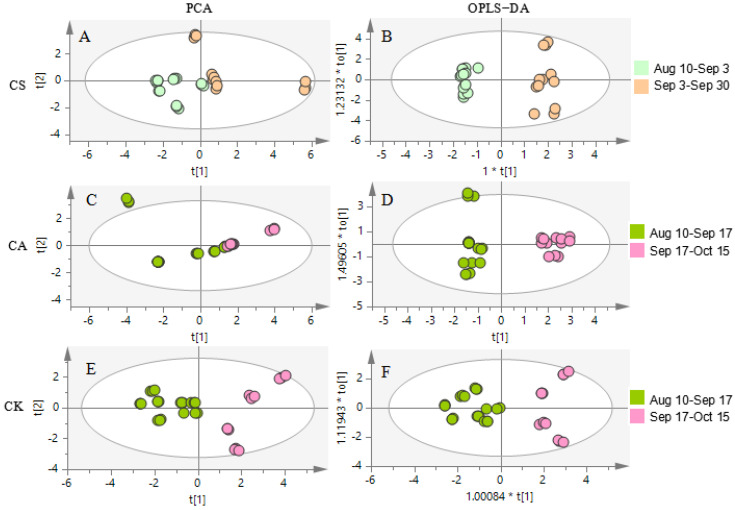
The statistical analysis of the content of eight flavonoids compounds score plot from PCA and OPLS−DA model with Hotelling’s 95% confidence ellipse of the leaves of CS, CA and CK in different harvesting period. (**A**) PCA in Aug 10–Sep 3 and Sep 3–Sep 30 of CS; (**B**) OPLS−DA in Aug 10–Sep 3 and Sep 3–Sep 30 of CS; (**C**) PCA in Aug 10–Sep 17 and Sep 17–Oct 15 of CA; (**D**) OPLS−DA in Aug 10–Sep 17 and Sep 17–Oct 15 of CA; (**E**)PCA in Aug 10–Sep 17 and Sep 17–Oct 15 of CK and (**F**) OPLS−DA in Aug 10–Sep 17 and Sep 17–Oct 15 of CK.

To further explore the similarity and difference of leaves in different harvesting periods, the PCA and OPLS−DA models were built as shown in Figure 9. According to the hierarchical clustering analysis (HCA) (Supporting information), and the Euclidean distance and Ward clustering algorithm were selected, the scores plot of PCA (R^2^X and Q^2^ (cum) were 0.984 and 0.893) in the harvesting period of CS was divided into two groups of harvesting period, which were from 10th August to 3th September (the blue color) and from 3rd September to 30th September (the gold color) shown in Figure 9A, the scores plot of OPLS−DA (R^2^X, R^2^Y and Q^2^ were 0.997, 0.981 and 0.953) had a good separation in Figure 9B, which demonstrated a marked difference in two groups. Combining S-plot of OPLS−DA and VIP, by filtering the compounds of VIP >1, we discovered rutin, quercetin, hyperoside, isoquercetin, vitexin and epicatechin were primarily markers in two harvesting periods. It suggested there were remarkable changes in August and September in the content of rutin, quercetin, hyperoside, isoquercetin, vitexin and epicatechin.

The same procedure was carried out separately for CA and CK, based on HCA (Supporting information), the scores plot of PCA for CA (Figure 9C) and CK (Figure 9E), CA and CK was divided into two same groups of harvesting period, which were from 10th August to 17th September (the green color) and from 17th September to 15th October (the pink color). The R^2^X and Q^2^ (cum) of the PCA model of CA were 0.984 and 0.905, and CK were 0.820 and 0.415 and the R^2^X, R^2^Y and Q^2^ of scores plot of OPLS−DA of CA (Figure 9D) were 1.000, 0.970 and 0.952, and CK (Figure 9F) were 0.795, 0.876 and 0.860. Combined S-plot of OPLS−DA and VIP, by filtering the compounds of VIP >1, we found quercetin, hyperoside, isoquercetin and epicatechin were common markers, which may be relevant with temperature [28].

#### 2.2.4. Comparison with the Content of Eight Flavonoids in Different Parts of CS and CK

**Figure 10:**
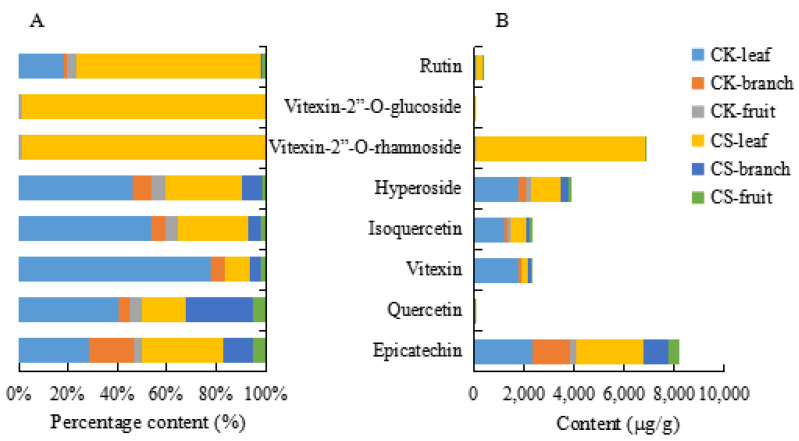
The content of eight flavonoids compounds in different parts of CS and CK. (**A**) Percentage stacked column of eight flavonoids compounds. (**B**) Stacked column of eight flavonoids compounds.

In the current study, samples of fruits, branches and leaves of CS and CK were picked on 17th September based on the best harvesting period of leaves. Detailed data were shown in Table S3. Vitexin-2”-*O*-rhamnoside and vitexin-2”-*O*-glucoside mainly existed in the leaf of CS, hyperoside, isoquercetin, vitexin and epicatechin mostly were detected in the leaf of CK. Unfortunately, vitexin-2”-*O*-rhamnoside and vitexin-2”-*O*-glucoside were not detected in the branches of CK and CS, and vitexin-2”-*O*-glucoside were not detected in the fruit of CS in Figure 10A. In addition, the content of hyperoside, isoquercetin, vitexin, rutin, quercetin and epicatechin were higher in leaves than branches and fruit in Figure 10B. Compared with different parts, we discovered the content of total flavonoids in leaves of CS and CK were 11.7 and 7.2 mg/g were highest. Moreover, the total flavonoids in branches of CS and CK were 1.6 and 2.0 mg/g, and the lowest content were 0.55 and 0.69 mg/g in the fruit of CS and CK in Table S3. Overall, the leaf as the primary part of food and medicinal material was in reason.

#### 2.2.5. The Content of Eight Flavonoids in the Leaves of CS and *C. pinnatifida*

**Figure 11:**
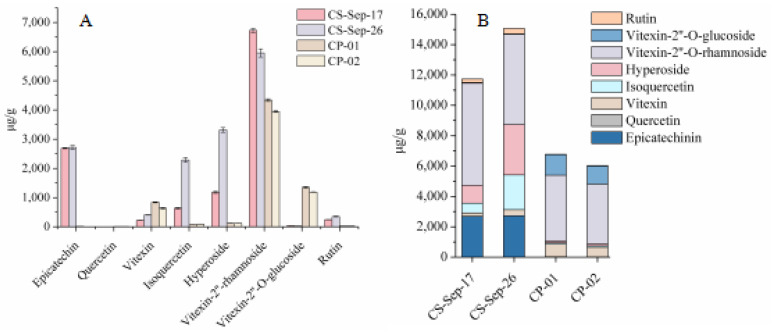
The content of eight flavonoids compounds in CS and CP. (**A**) Column of content of eight flavonoids compounds. (**B**) Stack column of content of eight flavonoids compounds.

In the current study, the collected leaves of CS on 17th and 26th September were selected to compare with *C. pinnatifida* (CP) according to the previous study. As shown in Figure 11A, the content of epicatechin, quercetin, isoquercetin, hyperoside, vitexin-2”-*O*-rhamnoside and rutin were higher in CS than CP, but the content of vitexin-2”-*O*-glucoside and vitexin were lower in CS than CP, what is more, the content of epicatechin in CS was about 270 times than in CP, hyperoside was almost 100 times, and isoquercetin and rutin were approximately 10 times. In addition, the total content of flavonoids in CS was higher than CP in Figure 11B, which suggested the leaves of CS were potential and valuable food and medicinal material.

## 3. Materials and Methods

### 3.1. Reagents and Materials

LC-MS grade acetonitrile and formic acid were purchased from Fisher Scientific (Loughborough, UK). HPLC grade methanol was obtained from Sigma-Aldrich (St. Louis, MO, USA). Deionized water (18.2 Ω) was further purified using a Milli-Q system from Millipore (Milford, MA, USA). Oasis^®^ PRiME HLB Cartridge Plus Short (335 mg) was provided by Waters Technologies (Milford, MA, USA). Eight flavonoids standards, rutin, quercetin, hyperoside, isoquercetin, vitexin-2”-*O*-glucoside, vitexin-2”-*O*-rhamnoside, vitexin, and epicatechin, were provided by Chengdu Must Bio-Technology CO., Ltd. (Chengdu, Sichuan, China). The purity of these standards was no less than 98%. All chemicals used for extraction were of analytical grade.

The leaves of different harvesting periods, fruit and branches of CS, CA and CK were collected from Xinjiang Academy of Forestry, Urumqi, Xinjiang, China and authenticated by Senior Scientist Hong Li (Xinjiang Academy of Forestry Sciences, Key Laboratory of Fruit tree Species Breeding and Cultivation in Xinjiang, Urumqi, China). The leaves of CS, CA and CK were collected once a week at the same time from August 10, 2020, until the leaves fell out. The leaves of CS were gathered in 9 batches of samples until September 30, 2020, CA and CK were in 11 batches until October 15, 2020. Additionally, the fruit and branches of CS and CK were picked in September 17, 2020. Moreover, *Crataegus pinnatifida* were purchased in Xiamen Shouyi Zhenyuan Health Management Co., Ltd. (Xiamen, Fujian, China).

### 3.2. UPLC–Q-TOF–MS Analysis

The instrument used was Acquity UPLC I-class system with an Acquity UPLC BEH C18 column (100 mm × 2.1 mm, 1.7 μm particles) from Wates Technologies (Milford, MA, USA) was used at 40 °C, and the samples were kept at 4 °C. A binary solvent system was employed consisting of solvent A (water containing 0.1% formic acid) and solvent B (acetonitrile). The gradient program was 0–10 min with 5–13% solvent B, 10–15 min with 13–20% B, 15–18 min with 20–50% B and 18–20 min with 50–95%B. The injection volume was 2 μL and the flow rate was set at 0.4 min/L.

Xevo G2-XS Q-TOF from Wates Technologies (Milford, MA, USA) equipped with electrospray ionization ion (ESI) source was applied for mass spectrometry data acquisition in negative ionization mode ranged from *m/z* 50−1200 Da in the profile mode. The lock mass compound was leucineenk-ephalin (*m/z* 554.2615), its concentration was 200 pg/mL. The ion scan mode adopted the MSE mode. The electrospray capillary voltage was set to 2.5 kV, the cone voltage was set to 40 V. The source temperature was 110 °C and desolvation temperature 450 °C. The low energy was 4 eV and the high energy ramp from 30 to 50 V. The cone gas flow was 50 L/h and desolvation gas flow 800 L/h; nitrogen and argon were used as the nebulizer and the collision gas respectively. The UPLC–Q-TOF–MS system was operated by MassLynx 4.1 software.

### 3.3. UPLC–TQ–MS Analysis

The instrument used was Acquity UPLC I-class system with an Acquity UPLC BEH C18 column (100 mm × 2.1 mm, 1.7 μm particles) from Wates Technologies (Milford, MA, USA) was used at 40 °C and the samples were kept at 4 °C. A binary solvent system was employed consisting of solvent A (water containing 0.1% formic acid) and solvent B (acetonitrile). The gradient program was 0–2 min with 15% solvent B, 2–5 min with 15–40% B and 5–6 min with 40–95% B. The injection volume was 2 μL and the flow rate was set at 0.4 mL/min.

Xevo TQ from Wates Technologies (Milford, MA, USA) equipped with the electrospray ionization ion (ESI) source was applied for mass spectrometry data acquisition in the negative ionization mode. The ion scan mode was adopted the MRM (multiple reaction monitoring) mode. The electrospray capillary voltage was set to 2.5 kV, the cone voltage was set to 40 V. The source temperature was 110 °C and desolvation temperature 450 °C. The cone gas flow was 50 L/h and desolvation gas flow 800 L/h; nitrogen and argon were used as the nebulizer and the collision gas respectively. The UPLC TQ MS system was controlled by MassLynx 4.1 software. The ions of quantitative analysis were monitored with rutin (609.3130→300.1526), quercetin (301.2661 → 151.0383), hyperoside (463.3189 → 300.1985), isoquercetin (463.3189 →271.0959), vitexin-2”-*O*-glucoside (593.3180 → 293.1144), vitexin-2”-*O*-rhamnoside (577.3231 → 293.1127), vitexin (431.3290 → 311.1557) and epicatechin (289.3025 → 245.1585).

### 3.4. Sample Preparation

Samples were dried to a constant weight under vacuum drying process, crushed in a ball crusher. Different samples accurately were weighed 250 mg and mixed with 5 mL methanol respectively. All the prepared samples were subjected to an ultrasonic bath for 30 min, cooled down, made up for weightlessness and then centrifuged for 5 min at 10,000 rpm with 4 °C. To Oasis ^®^ PRiME HLB Cartridge (SPE) 1 mL supernatant was added, and then 4 mL of methanol was eluted to a 5 mL volumetric flask and methanol was added to bring the volume to the scale and mixed thoroughly. All samples were filtered through a 0.22 μm syringe filter before analysis. Besides, three replicates were prepared for every sample. Then, a mixed quality control (QC) sample was prepared by pooled 100 μL of each sample for qualitative analysis, and the QC sample injections were used for the initial equilibration of the LC-MS system (4–5 injections) and one QC sample injected every 7 real sample injections during the list [26].

### 3.5. Quantitative Method Validation

In this paper, the 8 flavonoids compounds detected by the reference substance were quantitatively analyzed. Eight flavonoid standards were dissolved in methanol to prepare the mother liquor with the concentration of 1.0 mg/mL respectively, and then 8 mixed reference substances were progressively diluted to 10 μg /mL. Finally, 10 μg /mL was diluted to a suitable concentration step by step for injection. Into the UPLC TQ MS system for each batch of standard samples 2 μL was injected. The established method for quantitative analysis was validated by the linearity, limit of detection (LOD), limit of quantification (LOQ) accuracy, repeatability and recovery. The linear equation was performed with the peak area of reference as Y-axis and the concentration of reference substance as x-axis, the weighing was 1/x and the calibration curve of every compound was performed by at least five different concentrations. Some samples beyond the linear range, which were diluted to the linear range before evaluation.

### 3.6. Data Analysis

Qualitative data were analyzed by Progenesis QI (nonlinear dynamics, version 2.4), the parameters were conducted by default, except for data processing because only the 0.5–21 min range was selected. On the other hand, statistical analyses of the quantitative data were conducted by OriginPro 8.0 (OriginLab Corporation), and the PCA and OPLS−DA models were generated in SIMCA 13.0 (Umetrics AB).

## 4. Conclusions

Two hundred and sixteen compounds were identified and 28 unique compounds were screened in *Crataegi folium* from qualitative data, which could be used to distinguish different species of *Crataegi folium*. An efficient LC-MS/MS method was developed, validated and successfully implemented to the analysis of the content of eight flavonoid compounds within 6 min in CS, CA and CK, and their best harvesting period in leaves was optimal for effective utilization as medicinal and food materials. Additionally, as the *s* result of high content of vitexin-2”-*O*-rhamnoside in the leaves of CS, it provided a theoretical basis for CS further research and quality control.

## Figures and Tables

**Table 1:** Identification of the unique compound selected VIP > 1 from S-plot compared CS with CK.

NO	Compound	RT (min)	Adducts	Formula	Fragmentation Ions	Mass Error (ppm)	*m/z*
1	*α*-D-Gal-(→3)-*α*-D-Gal-OMe	0.64	M-H_2_O-H	C_13_H_24_O_11_	309(13), 191(100), 137(12), 125(7)	−2.53	337.1131
2	(2S)-Hex-2-ulofuranosyl-4,6-dideoxyhexopyranoside	0.96	M-H	C_12_H_22_O_9_	309(100), 179(62), 129(97), 119(54), 89(53)	2.92	309.1200
3	Glucogallin	1.24	M-H	C_13_H_16_O_10_	331(89), 168(49), 149(100), 125(61), 107(1)	6.54	331.0692
4	Eucomic acid	2.50	M+FA-H	C_11_H_12_O_6_	285(80), 208(13), 152(52), 121(1), 108(100)	3.75	285.0625
5	2-succinyl-6-hydroxycyclohexa-2,4-diene-1-carboxylic acid	4.01	M-H	C_11_H_12_O_6_	203(3), 179(7), 163(22), 119(100)	3.62	239.0573
6	(3R,5S,6S,7*E*,9S)-Megastigman-7-ene-3,5,6,9-tetrol-9-*O*-β-D-glucopyranoside	4.49	M+FA-H	C_19_H_34_O_9_	395(1), 167(28), 153(14), 145(100)	0.12	451.2185
7	Procyanidin B1	4.69	M-H	C_30_H_26_O_12_	451(10), 425(86), 407(100), 289(91), 245(39), 125(60)	−3.90	577.1354
8	Chlorogenic acid	5.52	M-H	C_16_H_18_O_9_	289(100), 205(18), 191(88), 123(51), 109(54)	2.39	353.0887
9	Procyanidin C1	7.00	M-H	C_45_H_38_O_18_	695(15), 577(23), 451(23), 289(99), 125(66)	4.79	865.2027
10	(Z)-3-Hexenyl-*O*-β-D-xylopyranosyl-(1”→6’)-β-D-glucopyranoside	8.02	M+FA-H	C_17_H_30_O_10_	221(1), 172(100), 131(2), 101(27), 89(8)	0.27	439.1822
11	Rutin	8.88	M-H	C_27_H_30_O_16_	563(8), 463(9), 446(81), 299(100), 271(10)	0.94	609.1467
12	2-*O*-{(2S,3R,4R)-4-[(*α*-L-arabinopyranosyloxy)methyl]-3,4-dihydroxytetrahydro-2-furanyl}-β-D-galactopyranoside	9.15	M-H_2_O-H	C_32_H_38_O_21_	739(100), 579(10), 569(30), 307(3)	−5.18	739.1688
13	7-*O*-rhamnogalactoside quercetin	9.48	M-H	C_27_H_30_O_16_	300(100), 271(17), 255(6), 243(1)	0.42	609.1464
14	6-*C*-glucoside-8-*C*-xylsoyl apigenin	10.00	M+FA-H	C_26_H_28_O_14_	500(6), 446(60), 251(100), 117(82), 89(3)	1.36	609.1469
15	vitexin-2”-*O*-rhamnoside	10.68	M-H	C_27_H_30_O_14_	413(20), 341(1), 311(4), 293(100), 269(1), 173(1)	0.30	577.1565
16	vitexin	10.70	M-H_2_O-H	C_21_H_20_O_10_	341(1), 311(8), 293(100), 269(1), 117(1)	4.34	413.0897
17	Vitexin-2”-*O*-glucoside	10.72	M-H	C_27_H_30_O_15_	577(44), 413(19), 341(1), 311(8), 293(100), 117(1)	0.66	593.1516
18	3-*O*-β-D-6”-acetylglucopyranoside quercetin	12.10	M-H	C_23_H_22_O_13_	463(1), 300(100), 271(51), 255(23), 243(6),151(5)	0.86	505.0992
19	Vitexin-3”-*O*-acetyl	13.04	M-H	C_23_H_22_O_11_	473(86), 413(23), 341(9), 311(96), 283(100), 161(9), 117(6)	1.01	473.1094
20	Picroside III	14.49	M-H	C_25_H_30_O_13_	462(2), 431(9), 316(1), 181(9), 153(100), 136(1)	−2.14	583.1674
21	Vitexin-6”-*O*-acetyl	15.53	M-H	C_23_H_22_O_11_	413(27), 311(68), 283(100), 268(4)	2.62	473.1102
22	Linalyl rutinoside	16.43	M-H	C_22_H_38_O_10_	413(26), 371(5), 211(7), 197(7), 145(100)	0.07	461.2393
23	Madecassic acid	18.31	M-H	C_30_H_48_O_6_	503(100), 485(17), 441(23), 235(1)	−0.73	549.3435
24	2α,3β,19α-thihydroxyl ursolic acid	19.14	M-H	C_30_H_48_O_5_	487(100), 469(8), 443(1), 423(1)	0.07	487.3429
25	3-*O*-*t*-p-Coumaroyltormentic acid	19.60	M-H	C_39_H_54_O_7_	633(100), 589(1),145(7), 119(1)	2.22	679.3878
26	(2α,3β)-2,19-Dihydroxy-3-{[(2*Z*)-3-(4-hydroxyphenyl)-2-propenoyl]oxy}urs-12-en-28-oic acid	19.81	M+FA-H	C_39_H_54_O_7_	633(100), 589(2),513(1), 145(16), 119(1)	2.98	679.3871
27	Jacoumaric acid	20.03	M-H	C_39_H_54_O_6_	617(100), 497(4), 472(49)	−1.09	617.3841
28	(3β,5ξ,9ξ,14β)-3-Hydroxy-27-{[(2*E*)-3-(4-hydroxyphenyl)-2-propenoyl]oxy}urs-12-en-28-oic acid	20.30	M-H	C_39_H_54_O_6_	617(100),573(1), 497(2), 453(1), 145(22),117(2)	1.56	617.3857

**Table 2:** Method validated data of the detected compounds.

Analytes	Calibration Curves	r	Linear Range (μg/g)	Accuracy (*n* = 6)	Repeatability (*n* = 6)	Recovery (*n* = 6)		LOD (μg/g)	LOQ (μg/g)
				RSD%	RSD%	Mean	RSD%		
Epicatechin	y = 9.79038x + 2.87783	0.9998	0.50–500	1.88	2.07	99.9	2.21	0.10	0.25
Quercetin	y = 42.0891x + 107.983	0.9990	0.10–100	1.96	1.83	100.9	1.71	0.05	0.10
Vitexin	y = 37.7813x − 29.4584	0.9997	0.50–500	1.30	3.78	99.4	3.17	0.25	0.50
Isoquercetin	y = 12.0094x − 36.7965	0.9997	0.50–500	1.99	3.73	99.4	3.56	0.25	0.50
Hyperoside	y = 23.6934x − 14.5733	0.9991	0.50–500	1.65	1.59	98.3	3.66	0.25	0.50
vitexin-2”-*O*-rhamnoside	y = 25.0249x − 91.4384	0.9997	0.50–500	1.26	1.51	98.1	2.43	0.25	0.50
Vitexin-2”-*O*-glucoside	y = 22.8099x − 59.7038	0.9991	0.50–500	1.69	3.06	100.5	3.06	0.10	0.25
Rutin	y = 24.1448x − 28.9144	0.9997	0.25–500	1.87	1.41	100.4	1.53	0.10	0.25

## Data Availability

Not applicable.

## Supplementary Materials

The following are available online, Figure S1: The HCA plot in different harvesting period of the leaves of CS (A), CA (B) and CK (C), Figure S2: The relative content of 28 unique compounds in the leaves of CS and CK in different harvesting periods, Table S1: 216 compounds information identified in the leaves of CS, CA and CK, Table S2: The content of eight flavonoids compounds of the leaves of CS, CA and CK, Table S3: The content of eight flavonoids compounds of different parts of CS and CK.

## Sample Availability

Not available.
