# Supplementary material for: Determination of Flavonoids Compounds of Three Species and Different Harvesting Periods in Crataegi folium Based on LC-MS/MS
Source: Molecules. 2021 Mar 13;26(6):1602. doi: 10.3390/molecules26061602 (PMC7999497; doi:10.3390/molecules26061602)
Supplement: Supplementary file 1 [file molecules-26-01602-s001.pdf]

Supplementary Data

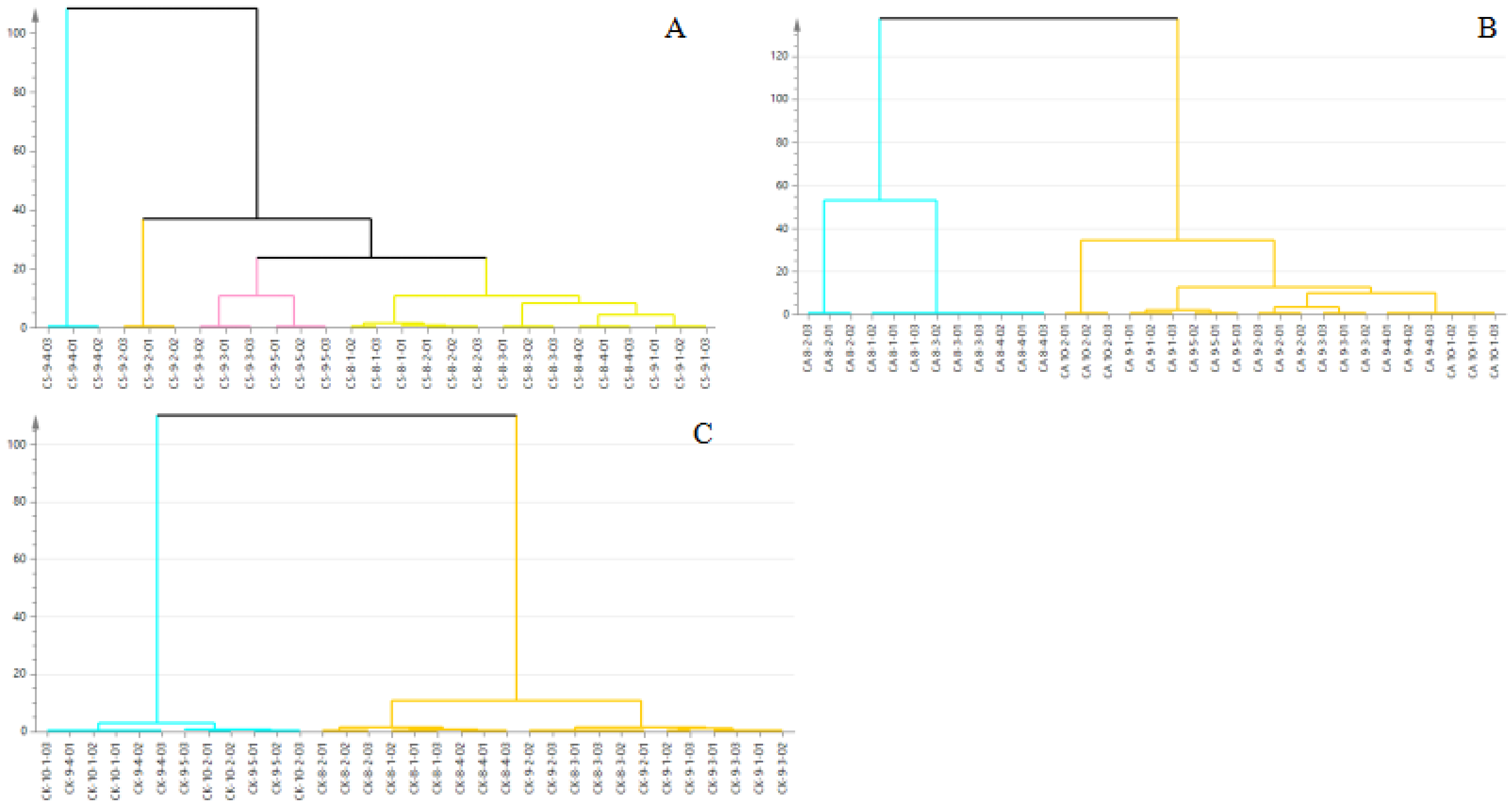

Fig. S1 The HCA plot in different harvesting period of the leaves of CS (A), CA(B), and CK (C).

Table S1 216 compounds information identified in the leaves of CS, CA and CK

NO	compound name	Retention time (min)	Adducts	Formula	Mass Error (ppm)	m/z
1	1-ethyl malate	0.61	M-H_2O-H	$C_6H_{10}O_5$	2.36	143.0354
2	papyriflavonol A	0.63	M+FA-H	$C_{25}H_{26}O_7$	7.46	483.1693
3	α-D-gal-(1→3)-α-D-gal-OMe	0.64	M-H_2O-H	$C_{13}H_{24}O_{11}$	-2.53	337.1131
4	6-O-galloyl-D-glucopyranose	0.66	M-H_2O-H	$C_{13}H_{16}O_{10}$	3.31	313.0576
5	Gallic acid	0.67	M-H	$C_7H_6O_5$	-4.61	169.0135
6	cis-Miyabenol C	0.79	M+FA-H	$C_{42}H_{32}O_9$	-2.42	725.2012
7	(2S,3S,4S,5R)-3,4,5-Trihydroxy-6-oxidotetrahydro-2H-pyran-2-carboxylate	0.80	M-H	$C_6H_8O_7$	0.18	191.0201
8	(2S)-Hex-2-ulofuranosyl-4,6-dideoxyhexopyranoside	0.96	M-H	$C_{12}H_{22}O_9$	2.92	309.1200
9	β-D-Glucopyranose	0.97	M-H	$C_6H_{12}O_6$	1.93	179.0565
10	4-Hydroxybenzoic acid	1.19	M-H	$C_7H_6O_3$	0.73	137.0245
11	7,3',4'-tri-O-methylluteolin	1.21	M-H	$C_{18}H_{16}O_6$	-4.10	327.0861
12	Glucogallin	1.24	M-H	$C_{13}H_{16}O_{10}$	6.54	331.0692
13	Benzoic acid	1.57	M+FA-H	$C_7H_6O_2$	2.61	167.0353
14	(4S)-4,5,8-trihydroxy-α-tetralone-4-O-β-D-glucopyranoside	1.63	M+FA-H	$C_{16}H_{20}O_9$	2.86	401.1100
15	Piscidic acid	1.63	M-H	$C_{11}H_{12}O_7$	4.40	255.0522
16	rhoiptelol B	1.70	M-H_2O-H	$C_{20}H_{24}O_6$	0.77	341.1397
17	(4R)-5,8-dihydroxy-4-methoxy-α-tetralone	2.09	M-H_2O-H	$C_{11}H_{12}O_4$	2.18	189.0562
18	Syringic acid	2.18	M-H_2O-H	$C_9H_{10}O_5$	2.79	179.0355
19	Veratric acid	2.32	M-H_2O-H	$C_9H_{10}O_4$	2.90	163.0406
20	eucomic acid	2.50	M+FA-H	$C_{11}H_{12}O_6$	3.75	285.0625

21	2-Propylsuccinic acid	2.65	M+FA-H	$C_7H_{12}O_4$	4.50	205.0725
22	4-hydroxybenzoic acid methyl ester	2.65	M-H	$C_8H_8O_3$	2.98	151.0405
23	3',7-O-diglucoside luteolin	2.70	M+FA-H	$C_{27}H_{30}O_{16}$	2.40	655.1530
24	3,4-Dihydroxybenzoic acid	2.87	M-H	$C_7H_6O_4$	2.12	153.0197
25	Ethyl 3-methoxy-4-hydroxybenzoate	2.89	M-H_2O-H	$C_{10}H_{12}O_4$	0.83	177.0559
26	p-methoxyphenylacetic acid	2.90	M-H_2O-H	$C_8H_8O_3$	0.64	133.0296
27	iso-orientin	3.09	M+FA-H	$C_{21}H_{20}O_{11}$	4.78	493.1009
28	(4S)-4,5,8-trihydroxy-α-tetralone 5-O-β-D-glucopyranoside	3.18	M+FA-H	$C_{16}H_{20}O_9$	2.17	401.1097
29	loliolide	3.23	M+FA-H	$C_{11}H_{16}O_3$	4.19	241.1090
30	5-Hydroxy-2-(4-hydroxyphenyl)-6-methoxy-4-oxo-4H-chromen-7-yl 6-O-[(2E)-3-(4-hydroxyphenyl)-2-propenoyl]hexopyranoside	3.33	M-H_2O-H	$C_{31}H_{28}O_{13}$	0.89	589.1357
31	Caffeic acid	3.57	M-H_2O-H	$C_9H_8O_4$	4.23	161.0252
32	6-O-acetyl-4-O-[6-O-acetyl-3-O-(2-carboxybenzoyl)-beta-D-glucopyranosyl]-2-O-(2-carboxybenzoyl)-beta-D-glucopyranose	3.58	M-H_2O-H	$C_{32}H_{34}O_{19}$	2.70	703.1535
33	Quinic acid	3.59	M-H_2O-H	$C_7H_{12}O_6$	3.51	173.0462
34	methyl 4-hydroxyphenylacetate	3.67	M-H	$C_9H_{10}O_3$	1.14	165.0559
35	4-Hydroxycinnamic acid	3.71	M-H	$C_9H_8O_3$	2.37	163.0405
36	Epipinoresinol	3.75	M+FA-H	$C_{20}H_{22}O_6$	-1.56	403.1393
37	5,7-dihydroxychromone	3.81	M-H	$C_9H_6O_4$	4.59	177.0201
38	3,4,5-Trimethoxyphenyl hexopyranoside	3.86	M+FA-H	$C_{15}H_{22}O_9$	3.50	391.1258
39	2-succinyl-6-hydroxycyclohexa-2,4-diene-1-carboxylic acid	4.01	M-H	$C_{11}H_{12}O_6$	3.62	239.0573
40	Pinnatifinoside F	4.01	M+FA-H	$C_{23}H_{20}O_{10}$	-0.89	501.1034
41	5-(β-D-Glucopyranosyloxy)-2-hydroxybenzoic acid	4.05	M-H_2O-H	$C_{13}H_{16}O_9$	4.36	297.0630
42	trans-coumaric acid methyl ester	4.05	M-H	$C_9H_8O_3$	1.42	163.0403
43	1-O-sinapoyl-β-D-glucose	4.11	M-H	$C_{17}H_{22}O_{10}$	4.64	431.1221

44	Poncirin	4.15	M+FA-H	$C_{28}H_{34}O_{14}$	-1.40	639.1922
45	herbacetin-3,8-diglucopyranoside	4.21	M+FA-H	$C_{27}H_{30}O_{17}$	4.36	671.1492
46	CSID28481414	4.23	M-H_2O-H	$C_{32}H_{38}O_{18}$	1.48	691.1890
47	broussonol E	4.26	M-H_2O-H	$C_{25}H_{26}O_7$	3.38	419.1515
48	4-(β-D-glucosyloxy)benzoic acid	4.32	M-H_2O-H	$C_{13}H_{16}O_8$	3.22	281.0676
49	1-(Hexopyranosyloxy)-1,4α,5,6,7,7α-hexahydrocyclopenta[c]pyran-7-carboxylic acid	4.41	M+FA-H	$C_{15}H_{22}O_9$	1.77	391.1252
50	(3R,5S,6S,7E,9S)-Megastigman-7-ene-3,5,6,9-tetrol-9-O-β-D-glucopyranoside	4.49	M+FA-H	$C_{19}H_{34}O_9$	0.12	451.2185
51	1-({6-O-[3,4-Dihydroxy-4-(hydroxymethyl)tetrahydro-2-furanyl]hexopyranosyl}oxy)-7-hydroxy-7-methyl-1,4a,5,6,7,7a-hexahydrocyclopenta[c]pyran-4-carboxylic acid	4.52	M-H_2O-H	$C_{21}H_{32}O_{14}$	0.22	489.1615
52	Procyanidin B1	4.69	M-H_2O-H, M-H	$C_{30}H_{26}O_{12}$	-3.90	577.1354
53	Vanillin	4.96	M-H_2O-H	$C_8H_8O_3$	1.53	133.0297
54	bis-(2-hydroxyethyl)terephthalate	4.96	M-H	$C_{12}H_{12}O_6$	0.90	297.0624
55	3,5-Dimethoxyethyl p-hydroxybenzoate	5.01	M-H_2O-H	$C_{11}H_{14}O_5$	2.16	207.0668
56	4α,5,8-trihydroxy-α-tetralone-5-O-β-D-[6'-O-(3",5"-dimethoxy-4"-hydroxybenzoyl)]glucopyranoside	5.01	M-H_2O-H	$C_{25}H_{28}O_{13}$	-2.18	517.1340
57	Syringetin 3-O-galactoside	5.02	M+FA-H	$C_{23}H_{24}O_{13}$	3.07	553.1215
58	Juglanosides F	5.06	M-H_2O-H	$C_{22}H_{28}O_{13}$	3.43	481.1369
59	1β,3α,12β,20(S)-tetrol-24-ene-dammar	5.23	M+FA-H	$C_{30}H_{52}O_4$	-2.17	521.3837
60	Troxerutin	5.25	M+FA-H	$C_{33}H_{42}O_{19}$	4.40	787.2335
61	Evofolin B	5.26	M+FA-H	$C_{17}H_{18}O_6$	-0.10	363.1085
62	Chlorogenic acid	5.52	M-H	$C_{16}H_{18}O_9$	2.39	353.0887
63	icariin	5.54	M+FA-H	$C_{33}H_{40}O_{15}$	-2.52	721.2332
64	p-Hydroxy-cinnamic acid	5.69	M-H	$C_9H_8O_3$	3.86	163.0407
65	methyl-4-Ocomaroylquinate	5.82	M+FA-H	$C_{17}H_{20}O_8$	4.03	397.1154
66	menadione	5.97	M+FA-H	$C_{11}H_8O_2$	2.73	217.0511

67	procyanidin B2	6.45	M-H_2O-H	$C_{30}H_{26}O_{12}$	1.44	559.1254
68	5'-O-(E-4-coumaroyl)-quinic acid	6.63	M-H_2O-H	$C_{16}H_{18}O_8$	3.64	319.0836
69	massonianoside D	6.95	M+FA-H	$C_{26}H_{34}O_{10}$	-0.62	551.2131
70	Procyanidin C1	7.00	M-H	$C_{45}H_{38}O_{18}$	4.79	865.2027
71	syringaresinol	7.17	M-H	$C_{22}H_{26}O_8$	-3.08	417.1542
72	mudanoside A	7.22	M-H_2O-H	$C_{14}H_{18}O_9$	4.30	311.0787
73	2,5-dihydroxy methyl phenylacetate	7.26	M-H_2O-H	$C_9H_{10}O_4$	2.60	163.0405
74	protocatechuic acid methyl ester	7.27	M-H	$C_8H_8O_4$	2.05	167.0353
75	4-O-β-D-glucopyranosylvanillc acid	7.28	M-H	$C_{14}H_{18}O_9$	4.04	329.0891
76	CSID5024	7.48	M-H	$C_{25}H_{22}O_{10}$	-3.20	481.1125
77	afzelechin	7.61	M-H	$C_{15}H_{14}O_5$	4.50	273.0781
78	3'-O-arabinosyl quercetin	7.84	M-H	$C_{20}H_{18}O_{11}$	2.27	433.0786
79	(4S)-4,6-dihydroxy-α-tetralone-4-O-β-D-glucopyranoside	7.86	M+FA-H	$C_{16}H_{20}O_8$	1.25	385.1144
80	Procyanidin	7.93	M-H_2O-H	$C_{30}H_{26}O_{13}$	3.85	575.1218
81	4-methoxy-α-tetralone-5-O-α-glucopyranoside	7.97	M+FA-H	$C_{17}H_{22}O_8$	2.02	399.1304
82	(Z)-3-Hexenyl-O-β-D-xylopyranosyl-(1"→6')-β-D-glucopyranoside	8.02	M+FA-H	$C_{17}H_{30}O_{10}$	0.27	439.1822
83	Neoeriocitrin	8.26	M+FA-H	$C_{27}H_{32}O_{15}$	4.70	641.1751
84	luteolin	8.32	M-H	$C_{15}H_{10}O_6$	4.56	285.0418
85	quercitrin	8.47	M-H	$C_{21}H_{20}O_{11}$	2.04	447.0942
86	breynioside A	8.53	M+FA-H	$C_{19}H_{20}O_9$	2.50	437.1099
87	(4S)-4-hydroxy-α-tetralone-4-O-β-D-glucopyranoside	8.73	M+FA-H	$C_{16}H_{20}O_7$	3.49	369.1202
88	1-O-vanilloyl-β-D-glucose	8.81	M-H_2O-H	$C_{14}H_{18}O_9$	4.18	311.0786
89	Rutin	8.88	M-H	$C_{27}H_{30}O_{16}$	0.94	609.1467
90	3-{[(2R,3S,4S)-3,4-Dihydroxy-4-(hydroxymethyl)tetrahydro-2-furanyl]oxy}-5-hydroxy-2-(4-hydroxyphenyl)-4-oxo-4H-chromen-7-yl 6-deoxy-alpha-L-mannopyranoside	8.91	M-H	$C_{26}H_{28}O_{14}$	0.05	563.1406

91	(2R)-2-(3,4-Dihydroxyphenyl)-7-hydroxy-4-oxo-3,4-dihydro-2H-chromen-5-yl 2-O-beta-D-glucopyranosyl-beta-D-glucopyranoside	9.10	M-H	$C_{27}H_{32}O_{16}$	-7.91	611.1569
92	2-(3,4-Dihydroxyphenyl)-5,7-dihydroxy-6-methoxy-4-oxo-4H-chromen-3-yl 2-O-{(2S,3R,4R)-4-[(alpha-L-arabinopyranosyloxy)methyl]-3,4-dihydroxytetrahydro-2-furanyl}-beta-D-galactopyranoside	9.15	M-H_2O-H	$C_{32}H_{38}O_{21}$	-5.18	739.1688
93	3-O-neohesperidoside kaempferol	9.19	M+FA-H	$C_{27}H_{30}O_{15}$	-2.11	639.1554
94	(7S,8R)-dihydrodehydrodiconiferyl alcohol 9-O-β-D-apiofuranosyl-O-β-D-glucopyranoside	9.19	M-H	$C_{31}H_{42}O_{15}$	0.89	653.2457
95	3-O-rhamnoside taxifolin	9.24	M-H	$C_{21}H_{22}O_{10}$	1.32	433.1146
96	Carlinoside	9.25	M-H	$C_{26}H_{28}O_{15}$	1.61	579.1365
97	1,3,5,6-tetrahydroxy-4,7,8-tri(3-methyl-2-butenyl)xanthone	9.36	M+FA-H	$C_{28}H_{32}O_{6}$	9.32	509.2224
98	7-O-rhamnogalactoside quercetin	9.48	M-H	$C_{27}H_{30}O_{16}$	0.42	609.1464
99	3,6-dihydroxy-4,5-dimethoxy-1,8-naphalic anhydride	9.49	M-H_2O-H	$C_{14}H_{10}O_{7}$	2.79	271.0256
100	eupatilin	9.64	M-H	$C_{18}H_{16}O_{7}$	1.95	343.0830
101	isoengeletin	9.65	M-H	$C_{21}H_{22}O_{10}$	0.18	433.1141
102	5,3'-O-diglucoside-eriodictyol	9.75	M-H_2O-H	$C_{27}H_{32}O_{16}$	0.80	593.1517
103	hesperetin	9.79	M-H	$C_{16}H_{14}O_{6}$	4.28	301.0731
104	myricetin-3-O-β-D-galactopyranoside	9.90	M-H	$C_{21}H_{20}O_{13}$	1.01	479.0836
105	6-C-glucosyl-8-C-xylsoyl apigenin	10.00	M+FA-H	$C_{26}H_{28}O_{14}$	1.36	609.1469
106	Caffeic acid methyl ester	10.01	M-H	$C_{10}H_{10}O_{4}$	3.79	193.0514
107	3,5-dihydroxy-1,4-naphthoquinone	10.06	M-H	$C_{10}H_{6}O_{4}$	0.90	189.0195
108	Jaceidin	10.06	M-H_2O-H	$C_{18}H_{16}O_{8}$	2.69	341.0676
109	juglone	10.06	M+FA-H	$C_{10}H_{6}O_{3}$	1.14	219.0301
110	eriodictyol	10.26	M-H_2O-H	$C_{15}H_{12}O_{6}$	0.68	269.0457
111	3-O-glucuronide-Quercetin	10.50	M-H	$C_{21}H_{18}O_{13}$	2.84	477.0688

112	isoquercitrin	10.58	M-H	$C_{21}H_{20}O_{11}$	2.65	447.0945
113	pinobanksin	10.58	M-H	$C_{15}H_{12}O_6$	3.36	287.0571
114	Vitexin-2"-O-rhamnosyl	10.68	M-H	$C_{27}H_{30}O_{14}$	0.30	577.1565
115	vitexin	10.70	M-H_2O-H	$C_{21}H_{20}O_{10}$	4.34	413.0897
116	Vitexin-2"-O-glucosyl	10.72	M-H	$C_{27}H_{30}O_{15}$	0.66	593.1516
117	6-C-xylsoyl-8-C-glucosyl apigenin	11.11	M-H	$C_{26}H_{28}O_{14}$	2.82	563.1422
118	colorless associated guinea	11.21	M-H	$C_{15}H_{14}O_6$	4.31	289.0730
119	Multiflorin B	11.23	M-H	$C_{27}H_{30}O_{15}$	1.48	593.1520
120	Avicularin	11.45	M-H	$C_{20}H_{18}O_{11}$	9.41	433.0817
121	chrysophanol	11.48	M+FA-H	$C_{15}H_{10}O_4$	1.57	299.0565
122	Vitexin-2"-O-(4-O-acetyl)-rhamnosyl	11.56	M-H	$C_{29}H_{32}O_{15}$	2.51	619.1684
123	1-O-galloyl-β-D-glucopyranose	11.87	M-H_2O-H	$C_{15}H_{20}O_{10}$	-4.66	341.0861
124	Coumarin	11.99	M-H	$C_9H_6O_2$	2.71	145.0299
125	pinnatifidin	12.00	M-H	$C_{21}H_{20}O_{11}$	-0.01	447.0933
126	3-O-β-D-6"-acetylglucopyranoside quercetin	12.10	M-H	$C_{23}H_{22}O_{13}$	0.86	505.0992
127	myricetin	12.10	M-H_2O-H	$C_{15}H_{10}O_8$	1.32	299.0201
128	6-O-feruloyl-D-glucopyranose	12.21	M-H	$C_{16}H_{20}O_9$	3.78	355.1048
129	ferulic acid	12.30	M-H	$C_{10}H_{10}O_4$	3.51	193.0513
130	4-hydroxy-4-(3'-hydroxyphenyl) butyric acid-4-O-β-D-glucopyranoside methyl ester	12.34	M-H_2O-H	$C_{17}H_{24}O_9$	1.23	353.1247
131	4-[6-O-(4-hydroxy-3,5-dimethoxybenzoyl)-β-D-glucopyranosyloxy]-3-methoxybenzoic acid	12.38	M-H_2O-H	$C_{23}H_{26}O_{13}$	3.18	491.1211
132	1,3-O-dicaffeoylquinic acid	12.44	M-H	$C_{25}H_{24}O_{12}$	2.20	497.1101
133	juglone A	12.50	M-H	$C_{12}H_{14}O_3$	3.89	205.0878
134	3,7,8,3'-tetrahydroxy-4'-methoxyflavone	12.66	M-H_2O-H	$C_{16}H_{12}O_7$	3.81	297.0417
135	3-Methylkaempferol	12.66	M-H_2O-H	$C_{16}H_{12}O_6$	3.12	281.0465

136	1,3-dihydroxy-2-methylanthraquinone	12.67	M-H	$C_{15}H_{10}O_5$	3.84	269.0466
137	2-hydroxy-3-methylanthraquinone	12.67	M+FA-H	$C_{15}H_{10}O_3$	1.97	283.0617
138	Vitexin-2"-O-acetyl	12.91	M+FA-H	$C_{23}H_{22}O_{11}$	1.82	519.1153
139	isovitexin	13.03	M-H	$C_{21}H_{20}O_{10}$	3.46	431.0999
140	Vitexin-3"-O-acetyl	13.04	M-H	$C_{23}H_{22}O_{11}$	1.01	473.1094
141	Wogonin	13.04	M-H	$C_{16}H_{12}O_5$	4.41	283.0624
142	naringenin	13.10	M-H	$C_{15}H_{12}O_5$	3.38	271.0621
143	Pinnatifinoside E	13.10	M+FA-H	$C_{23}H_{20}O_{10}$	1.51	501.1045
144	3-O-glucoside-8-methoxy kaempferol	13.18	M-H	$C_{22}H_{22}O_{12}$	1.72	459.0941
145	(+)catechin	13.23	M-H	$C_{15}H_{14}O_6$	3.80	289.0729
146	SZY-7	13.23	M-H	$C_{30}H_{26}O_{11}$	1.39	561.1410
147	3-O-glucoside-syringetin	13.54	M-H_2O-H	$C_{23}H_{24}O_{13}$	4.19	489.1060
148	1-hydroxy-2-methyl-4-methoxyanthraquinone	13.58	M-H_2O-H	$C_{16}H_{12}O_4$	2.81	249.0565
149	Afzelin	13.77	M+FA-H	$C_{21}H_{20}O_{10}$	6.08	477.1065
150	8-C-glucosyl-rhamnosyl apigenin	13.90	M-H	$C_{27}H_{30}O_{14}$	-0.36	577.1561
151	4'-hydroxy-2'-methoxyphenol-1-O-β-D-(6-O-syringoyl)glucopyranoside	13.92	M-H_2O-H	$C_{23}H_{28}O_{11}$	4.39	461.1474
152	1-O-Benzoyl-alpha-D-glucopyranose	14.00	M-H_2O-H	$C_{13}H_{16}O_7$	3.61	265.0728
153	1-O-(4-coumaroyl)-beta-D-glucose	14.01	M-H	$C_{15}H_{18}O_8$	4.52	325.0938
154	4-(1-hydroxy-1-methylethyl)-benzoic acid	14.01	M-H_2O-H	$C_{10}H_{12}O_3$	-1.93	161.0605
155	quercetin-3-O-(4"-O-acetyl)-α-L-rhamnopyranoside	14.18	M-H	$C_{23}H_{22}O_{12}$	3.55	489.1056
156	(5R,6S,7S,8S)-8-Acetoxy-6-hydroxy-1,2,3,13-tetramethoxy-6,7-dimethyl-5,6,7,8-tetrahydrobenzo[3',4']cycloocta[1',2':4,5]benzo[1,2-d][1,3]dioxol-5-yl(2Z)-2-methyl-2-butenoate	14.38	M-H	$C_{30}H_{36}O_{11}$	2.61	571.2200
157	Picroside III	14.49	M-H	$C_{25}H_{30}O_{13}$	-2.14	583.1674
158	Hendecanoic acid	14.51	M+FA-H	$C_{11}H_{22}O_2$	3.94	231.1609
159	Pinnatifinoside C	14.56	M-H	$C_{23}H_{20}O_{10}$	1.99	455.0993

160	(6S,7E,9R)-6,9-Dihydroxy-4,7-megastigmadien-3-one-9-O-β-D-glucopyranoside	14.58	M-H	$C_{19}H_{30}O_{8}$	-2.02	385.1860
161	Pinnatifinoside A	14.60	M+FA-H	$C_{21}H_{18}O_{9}$	3.26	459.0946
162	1,4,8-trihydroxynaphthalenel-O-β-D-[6'-O-(4"-hydroxy-3",5"-dimethoxybenzoyl)]glucopyranoside	14.61	M-H$_2$O-H	$C_{25}H_{26}O_{12}$	2.15	499.1257
163	Pinnatifinoside B	14.84	M-H	$C_{23}H_{20}O_{10}$	4.82	455.1006
164	taxifolin	14.86	M-H$_2$O-H	$C_{15}H_{12}O_{7}$	0.66	285.0407
165	schizandriside	14.89	M+FA-H	$C_{25}H_{32}O_{10}$	1.16	537.1983
166	Benzyl 2-(hexopyranosyloxy)-6-hydroxybenzoate	14.94	M-H	$C_{20}H_{22}O_{9}$	2.48	405.1201
167	(4R)-4-hydroxy-α-tetralone	15.04	M-H	$C_{10}H_{10}O_{2}$	-3.91	161.0602
168	5-O-rhamnosyl apigenin	15.15	M+FA-H	$C_{21}H_{20}O_{9}$	2.16	461.1098
169	glogoside	15.22	M-H$_2$O-H	$C_{22}H_{22}O_{12}$	3.29	459.0949
170	schaftosise	15.27	M-H$_2$O-H	$C_{26}H_{28}O_{14}$	4.64	545.1327
171	Pinnatifinoside D	15.41	M-H	$C_{23}H_{20}O_{10}$	0.94	455.0988
172	juglanone	15.42	M-H$_2$O-H	$C_{19}H_{16}O_{8}$	2.85	353.0677
173	7-O-glucoside apigenin	15.53	M-H$_2$O-H	$C_{21}H_{20}O_{10}$	2.15	413.0887
174	tricin	15.53	M-H$_2$O-H	$C_{17}H_{14}O_{7}$	3.42	311.0572
175	Vitexin-6"-O-acetyl	15.53	M-H	$C_{23}H_{22}O_{11}$	2.62	473.1102
176	1-hydroxy-6-methyl-anthraquinone	15.53	M+FA-H	$C_{15}H_{10}O_{3}$	4.39	283.0622
177	3-O-rhamnosyl quercetin	15.58	M-H	$C_{21}H_{20}O_{11}$	-0.01	447.0933
178	6"-O-vanilloylisotachioside	15.59	M-H$_2$O-H	$C_{21}H_{24}O_{11}$	3.87	433.1158
179	Hyperoside	15.60	M-H$_2$O-H	$C_{21}H_{20}O_{12}$	2.36	445.0787
180	2'-hydroxy-4'-methoxyphenol-1-O-β-D-(6-O-syringoyl)glucopyranoside	15.63	M-H$_2$O-H	$C_{23}H_{28}O_{11}$	-2.64	461.1441
181	5-Hydroxy-3,7-dimethoxy-3',4'-methylenedioxyflavone	15.78	M-H	$C_{18}H_{14}O_{7}$	-1.89	341.0675
182	5-p-cis-coumaroylquinic acid	15.82	M-H	$C_{16}H_{18}O_{8}$	3.38	337.0940
183	engeletin	15.97	M-H$_2$O-H	$C_{21}H_{22}O_{11}$	1.55	431.0991

184	2-cis-4-trans-abscisic acid	16.04	M-H	$C_{15}H_{20}O_4$	4.59	263.1301
185	apigenin	16.06	M-H	$C_{15}H_{10}O_5$	0.55	269.0457
186	(6R,9R)-9-Hydroxymegastigman-4-en-3-one-9-O-β-D-glucopyranoside	16.14	M-H	$C_{19}H_{32}O_7$	3.43	371.2088
187	4-[7-(β-D-Glucopyranosyloxy)-5-hydroxy-3-({2-O-[(2E)-3-(4-hydroxy-3-methoxyphenyl)-2-propenoyl]-beta-D-glucopyranosyl}oxy)-4-oxo-4H-chromen-2-yl]phenyl beta-D-glucopyranoside	16.21	M+FA-H	$C_{43}H_{48}O_{24}$	2.22	993.2539
188	5-methoxy-1,4-naphthoquinone	16.31	M-H	$C_{11}H_8O_3$	-1.24	187.0398
189	Linalyl rutinoside	16.43	M-H	$C_{22}H_{38}O_{10}$	0.07	461.2393
190	2,3-dihydro-2-(4'-O-β-D-glucopyranosyl-3'-methoxy-phenyl)-3-hydroxymethyl-5-(3-hydroxypropyl)-7-methoxybenzofuran	16.52	M+FA-H	$C_{26}H_{34}O_{11}$	-4.58	567.2059
191	cantonienol	16.64	M+FA-H	$C_{15}H_{26}O_3$	3.34	299.1872
192	Pinnatifinoside B	16.71	M-H	$C_{23}H_{20}O_{10}$	1.51	455.0991
193	4-(5,7-Dihydroxy-4-oxo-4H-chromen-2-yl)phenyl 6-O-[(2E)-3-(4-hydroxyphenyl)-2-propenoyl]-beta-L-glucopyranoside	16.71	M-H	$C_{30}H_{26}O_{12}$	3.36	623.1419
194	Dihydrokaempferol 3-O-rhamnoside	16.72	M-H	$C_{21}H_{20}O_{10}$	0.32	413.0879
195	3,4'-dihydroxy-5,6,7-trimethoxyflavones	16.85	M-H_2O-H	$C_{18}H_{16}O_7$	1.90	325.0724
196	kakispyrol	16.89	M-H	$C_{20}H_{24}O_9$	4.12	407.1364
197	(1'R,3'R,5'R,8'S)-epi-dihydrophaseic acid-β-D-glucoside	16.92	M-H_2O-H	$C_{21}H_{32}O_{10}$	1.26	425.1823
198	4-[(4R-4-(β-D-glucopyranosyloxy)-2,6,6-Trimethyl-1-cyclohexen-1-xl]-2-butanone	17.00	M+FA-H	$C_{19}H_{32}O_7$	3.62	417.2144
199	3-deoxidization-isoquercetin	17.36	M-H	$C_{22}H_{22}O_{10}$	-2.89	445.1127
200	Erythro-guaiacylglycerol-β-O-4'-coniferyl alcohol	17.41	M+FA-H	$C_{20}H_{24}O_7$	2.61	421.1514
201	roseoside	17.44	M-H_2O-H	$C_{19}H_{30}O_8$	4.11	367.1778
202	4-(4-Hydroxy-2,6,6-trimethyl-1-cyclohexen-1-yl)-2-butanyl 6-O-[3,4-dihydroxy-4-(hydroxymethyl)tetrahydro-2-furanyl]hexopyranoside	17.67	M-H_2O-H	$C_{24}H_{42}O_{11}$	2.12	487.2559
203	Myristic acid	17.72	M-H	$C_{14}H_{28}O_2$	1.47	227.2020

204	Madecassic acid	18.31	M-H	$C_{30}H_{48}O_6$	-0.73	549.3435
205	11-hydroxy-2,4-cycloeudesman-8-one	18.34	M+FA-H	$C_{15}H_{24}O_2$	4.22	281.1768
206	10,11-Dihydroxy-2,2,6α,6β,12α-pentamethyl-9-methylene-1,3,4,5,6,6α,6β,7,8,8α,9,10,11,12,12α,12β,13,14β-octadecahydro-2H-picene-4a-carboxylic acid	18.41	M+FA-H	$C_{29}H_{44}O_4$	0.19	501.3223
207	2α,3β,19α-thihydroxyl ursolic acid	19.14	M-H	$C_{30}H_{48}O_5$	0.07	487.3429
208	2-[({(3β,5α,6β)-3-[(3-Carboxypropanoyl)oxy]-5-hydroxycholestan-6-yl}oxy)carbonyl]benzoic acid	19.34	M-H_2O-H	$C_{39}H_{56}O_9$	2.31	649.3762
209	3-O-trans-p-Coumaroyltormentic acid	19.60	M-H	$C_{39}H_{54}O_7$	2.22	679.3878
210	(2α,3β)-2,19-Dihydroxy-3-{[(2Z)-3-(4-hydroxy-3-methoxyphenyl)-2-propenoyl]oxy}urs-12-en-28-oic acid	19.69	M-H	$C_{40}H_{56}O_8$	-2.69	663.3885
211	(2α,3β)-2,19-Dihydroxy-3-{[(2Z)-3-(4-hydroxyphenyl)-2-propenoyl]oxy}urs-12-en-28-oic acid	19.81	M+FA-H	$C_{39}H_{54}O_7$	2.98	679.3871
212	(3β,5x)-3-Hydroxy-27-{[(2E)-3-(4-hydroxyphenyl)-2-propenoyl]oxy}urs-12-en-28-oicacid	20.03	M+FA-H	$C_{39}H_{54}O_6$	4.35	663.3929
213	Jacoumaric acid	20.03	M-H	$C_{39}H_{54}O_6$	-1.09	617.3841
214	Ulmicin E	20.22	M-H_2O-H	$C_{45}H_{60}O_7$	5.94	693.4203
215	(3β,12β,20Z)-12-Hydroxydammara-20(22),24-dien-3-yl 2-O-(6-O-acetyl-beta-D-glucopyranosyl)-beta-D-glucopyranoside	20.27	M+FA-H	$C_{44}H_{72}O_{13}$	-8.43	853.4887
216	(3β,5x,9x,14β)-3-Hydroxy-27-{[(2E)-3-(4-hydroxyphenyl)-2-propenoyl]oxy}urs-12-en-28-oic acid	20.30	M-H	$C_{39}H_{54}O_6$	1.56	617.3857

Table S2 The the content of eight flavonoids compounds of the leaves of CS, CA and CK

NO	Harvesting Date	Epicatechin	Quercetin	Vitexin	Isoquercetin	Hyperoside	Vitexin-2"-O-rhamnosyl	Vitexin-2"-O-glucosyl	Rutin
		μg/g (n=3, mean ± SD)							
CS-8-1	August 10, 2020	808.64±27.03	0.53±0.01	148.18±3.9	133.68±3.14	286.24±7.84	6914.98±193.59	40.22±1.04	145.46±3.11
CS-8-2	August 13, 2020	638.85±2.92	0.48±0.01	192.38±2.71	73.81±2.42	203.72±7.60	7276.68±33.27	40.69±0.49	151.27±3.16
CS-8-3	August 20, 2020	621.15±12.43	1.08±0.02	142.14±2.66	172.54±2.87	353.83±6.42	6332.8±61.99	34.16±0.59	190.49±5.37
CS-8-4	August 27, 2020	1390.47±42.83	1.72±0.03	291.71±1.71	643.34±21.43	909.41±1.75	6744.34±12.26	36.48±0.63	193.79±1.54
CS-9-1	September 03, 2020	1100.78±30.07	0.38±0.01	230.00±3.11	106.06±3.90	212.26±6.94	7085.72±152.92	38.57±1.16	219.80±4.96
CS-9-2	September 10, 2020	2185.15±31.07	1.08±0.02	342.78±7.47	497.06±12.79	916.98±22.37	8192.38±107.94	45.82±1.02	269.55±7.07
CS-9-3	September 17, 2020	2691.45±17.04	1.85±0.06	223.47±4.67	634.82±17.87	1180.74±34.08	6728.38±73.28	37.96±1.20	242.66±6.23
CS-9-4	September 26, 2020	2718.77±61.36	4.90±0.11	402.51±7.18	2290.16±77.33	3315.03±79.53	5953.62±140.67	31.35±0.90	348.83±11.18
CS-9-5	September 30, 2020	794.64±24.32	3.08±0.07	267.66±8.56	987.18±14.99	1842.85±47.58	6632.23±185.67	39.24±0.32	223.06±5.57
CK-8-1	August 10, 2020	1473.75±20.15	2.34±0.08	1994.24±27.9	570.66±20.19	873.72±6.40	1.25±0.050	ND	35.63±1.63
CK-8-2	August 13, 2020	1752.55±28.59	2.43±0.05	2302.84±85.74	679.57±16.10	1064.49±25.86	66.08±0.50	ND	25.89±1.13
CK-8-3	August 20, 2020	2139.34±7.60	2.71±0.05	2555.83±15.21	705.83±28.33	1014.45±16.32	1.14±0.04	ND	29.86±1.26
CK-8-4	August 27, 2020	1832.94±35.94	1.74±0.03	1845.95±33.52	407.95±4.52	628.79±17.45	23.2±0.78	ND	18.00±0.33
CK-9-1	September 03, 2020	2712.82±97.82	2.32±0.05	2300.34±93.74	968.22±151.14	1331.66±40.95	0.95±0.02	ND	73.82±6.46
CK-9-2	September 10, 2020	2598.81±26.78	1.74±0.05	2022.95±47.76	491.51±7.73	728.84±16.45	25.76±1.05	ND	92.26±1.88
CK-9-3	September 17, 2020	2350.38±49.03	4.26±0.05	1783.82±9.00	1200.94±42.46	1756.68±38.01	ND	ND	60.24±0.79

CK-9-4	September 26, 2020	4204.71±42.47	3.69±0.09	2465.48±26.8	1262.96±30.03	1732.42±20.9	1.77±0.03	ND	133.03±1.82
CK-9-5	September 30, 2020	4307.55±80.09	4.25±0.09	2505.88±67.59	1136.46±25.82	1380.93±22.66	ND	ND	259.94±3.41
CK-10-1	October 08, 2020	2946.65±57.97	8.13±0.33	2611.04±93.77	1651.57±33.38	2401.79±18.23	ND	ND	102.11±2.67
CK-10-2	October 15, 2020	2518.4±35.63	10.67±0.07	2981.61±75.07	2164.78±72.96	2963.52±67.38	ND	ND	86.73±2.26
CA-8-1	August 10, 2020	56.06±0.26	0.00±0.00	42.29±1.11	9.49±0.26	17.41±0.09	ND	ND	0.63±0.01
CA-8-2	August 13, 2020	16.23±0.55	0.00±0.00	18.96±0.59	8.50±0.10	13.56±11.75	300.52±10.73	2.50±0.11	4.11±0.15
CA-8-3	August 20, 2020	112.93±1.70	0.00±0.00	62.72±1.71	9.48±0.19	16.96±0.27	ND	ND	0.90±0.02
CA-8-4	August 27, 2020	113.98±3.74	0.00±0.00	63.00±2.19	2.71±0.15	4.81±0.17	ND	ND	0.00±0.00
CA-9-1	September 03, 2020	3000.74±48.07	1.54±0.04	2914.44±36.21	543.85±14.33	791.33±21.79	0.93±0.04	ND	114.88±2.04
CA-9-2	September 10, 2020	2490.39±31.07	1.54±0.01	1279.81±61.52	444.52±17.05	633.17±28.69	0.62±0.01	ND	18.15±0.11
CA-9-3	September 17, 2020	4043.36±44.86	0.00±0.00	1898.88±15.99	762.48±21.8	1036.58±3.13	0.62±0.01	ND	34.76±1.35
CA-9-4	September 26, 2020	3663.53±94.73	3.09±0.05	1677.74±23.28	1070.91±24.83	1350.12±2.61	0.57±0.02	ND	31.45±1.00
CA-9-5	September 30, 2020	4360.72±81.77	2.99±0.04	1842.8±28.48	742.47±5.74	955.75±17.60	ND	ND	121.54±4.98
CA-10-1	October 08, 2020	3353.22±49.96	4.26±0.03	1743.5±48.91	1061.52±35.96	1359.12±18.96	ND	ND	29.34±0.93
CA-10-2	October 15, 2020	2972.34±53.56	6.75±0.15	2286±61.85	1472.27±26.68	2169.16±53.13	ND	ND	179.76±6.12

* ND not detected

Table S3 The the content of eight flavonoids compounds of different parts of CS and CK

NO	Epicatechin	Quercetin	Vitexin	Isoquercetin	Hyperoside	Vitexin-2"-O-rhamnosyl	Vitexin-2"-O-glucosyl	Rutin	Summary content
	μg/g (n=3, mean ± SD)								
CK-leaf	2350.38±49.03	4.26±0.05	1783.82±9.00	1200.94±42.46	1756.68±38.01	ND	ND	60.24±0.79	7156.31±139.33
CS-leaf	2691.45±17.04	1.85±0.06	223.47±4.67	634.82±17.87	1180.74±34.08	6728.38±73.28	37.96±1.20	242.66±6.23	11741.31±28.37
CK-branch	1455.87±16.92	0.48±0.08	117.91±3.05	130.23±3.65	288.81±4.53	ND	ND	3.76±0.13	1997.07±27.99
CS-branch	1024.65±36.29	2.93±0.24	101.88±3.35	126.09±11.2	301.64±11.81	ND	ND	3.24±0.09	1560.43±154.44
CK-fruit	269.64±12.42	0.56±0.08	10.85±0.32	100.74±5.23	219.99±7.85	74.56±1.62	0.34±0.03	11.7±0.44	688.39±62.98
CS-fruit	405.99±13.26	0.54±0.03	50.58±1.89	39.36±2.20	53.3±1.70	0.10±0.02	ND	4.33±0.31	554.19±19.41

* ND not detected
